# Effects of motor imagery training on generalization and retention for different task difficulties

**DOI:** 10.3389/fnhum.2024.1459987

**Published:** 2024-10-16

**Authors:** Yoichiro Sato

**Affiliations:** Department of Physical Therapy, Faculty of Health Sciences, Hokkaido University of Science, Sapporo, Hokkaido, Japan

**Keywords:** mental training, generalization, retention, different task difficulties, motor learning

## Abstract

Although previous studies have suggested that motor adaptation through motor imagery training of similar tasks can improve retention and generalization of motor learning, the benefits of mental and physical training remain unclear for different task difficulties. Two experiments were conducted in this study. The first experiment aimed to determine whether there were differences in movement time (MT) when drawing circles based on three conditions in accordance with Fitts’ law. The results showed significant differences in MT among the three conditions (*p* < 0.001), with MT becoming long as the width of the circle line (which indicated different difficulty level) narrowed. The second experiment aimed to determine whether the task difficulty influenced immediate generalization and retention at 24 h after mental vs. physical training. Participants in both training groups practiced the task with the medium-sized circle, which indicated medium difficulty. The posttest results revealed that mental training leads to considerable performance improvement than physical training, as demonstrated by a shorter MT regardless of the task difficulty level. Meanwhile, the retention test results showed no difference in generalization between mental and physical training. However, generalization of an easier task was more effectively retained than more difficult tasks. These results suggest that mental training can improve performance during the adaptation phase and that difficulty level can influence the degree of retention.

## 1 Introduction

Sensorimotor adaptation is essential for skill acquisition and allows us to adapt flexibly to new and challenging environments. By repeated training, movements become faster and more accurate (Ruffino et al., 2021; Willingham, 1998). Although physical training is fundamental to acquire new skills, motor imagery training (MIT) is a complementary method (Mulder et al., 2007). MIT is defined as the mental simulation of movement without actual physical outputs (Ruffino et al., 2021). MIT can improve movement flexibility (Kanthack et al., 2017), as well as speed and accuracy (Allami et al., 2008; Gentili et al., 2010), as a potential method for motor rehabilitation (Jackson et al., 2001; Dickstein et al., 2014). The positive impact of MIT on motor learning is associated with neural adaptations at several levels within the central nervous system (Avanzino et al., 2015).

As most motor behaviors in changeable environments have not been previously experienced, practiced performances must be generalized or transferred, or both to tasks without practice. Moreover, improved performance requires generalization of other tasks with different levels of difficulty. Gentili et al. (2006) reported that MIT can generalize from movement trajectories of the trained right arm to the untrained left arm. In addition, the degree of generalization of MIT is reportedly similar to that applied in physical training. Conessa et al. (2023) reported that daytime napping promoted the consolidation and retention of learned motor skills following mental and physical training. These reports indicate that mental and physical training can stimulate generalization, consolidation, and retention after sleeping. However, although previous studies demonstrated that the influence of mental and physical trainings on generalization and retention of tasks with the same levels of difficulty, the influence on generalization and retention of tasks with different levels of difficulty remains unclear.

Therefore, two experiments were conducted in this study to determine (i) whether there are differences in the immediate effects of mental and physical training on performance, (ii) whether immediate generalization is influenced by the two modalities, and (iii) whether the degrees of retention and generalization differ between the two modalities. The aim of experiment 1 was to determine whether the tasks selected in this study exhibited different movement times (MTs) due to differences in the level of difficulty, such as the reciprocal tapping task, as described by the Fitts (1954), where the participants tapped back and forth between two rectangular targets. The aim of experiment 2 was to examine the influence of mental and physical training on generalization and retention using the tasks of experiment 1. In experiment 2, the participants were instructed to perform a task using the nondominant left arm, as all of the participants were right-handed. Reportedly, as the trajectories of dominant and nondominant hands show asymmetry, the right and left cerebral hemispheres differentially mediate the control of each arm (Sainburg, 2002; Sainberg and Wang, 2002). The right hemisphere of the brain, which controls the non-dominant hand, relies on peripheral feedback-mediated impedance control mechanisms (Mutha et al., 2012). Conversely, the left hemisphere of the brain plays an important role in feed-forward control processes (Mutha et al., 2012). Thus, differences between mental and physical training should be easily detected because performing a relatively difficult task with the nondominant hand requires highly accurate feedback information. Therefore, immediate generalization of a relatively difficult task should be improved after physical training vs. mental training because of the lack of feedback. Moreover, retention should be lower after mental training than physical training because more difficult tasks require greater feedback.

## 2 Materials and methods

### 2.1 Experiment 1

#### 2.1.1 Study approval and patient consent

The study protocol was approved by the Institutional Review Committee of Hokkaido University of Science (approval no. 375) and conducted in accordance with the ethical principles for medical research involving human subjects described in the Declaration of Helsinki. Prior to inclusion in this study, informed consent was obtained from all participants.

#### 2.1.2 Participants

The study cohort for experiment 1 included 40 healthy young adults (19 women and 21 men; mean age, 21.5 ± 2.8 years). All participants were right-handed without developmental, neurological, mental, or physical disorders.

#### 2.1.3 Circle-drawing task

**Figure 1:**
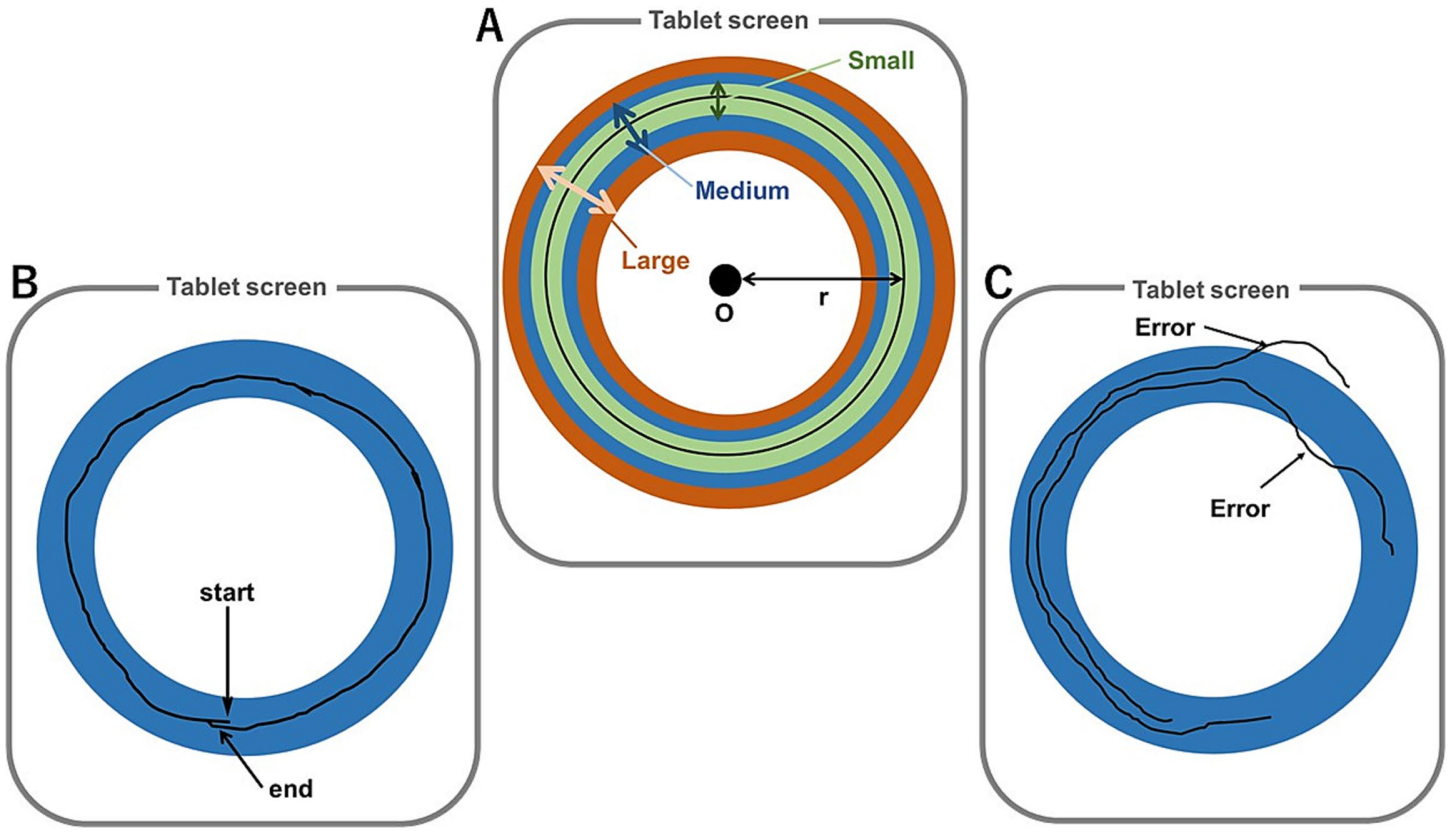
A schematic of the circle-drawing task. **(A)** Image of the task on a tablet. The radius (r) of the large, medium, and small circles. **(B)** Successful trial. All practices were performed with the medium-sized circle [blue line in panel **(A)**]. **(C)** Examples of errors (not counted). A drawing beyond the line.

The participants were instructed to draw circles using the right hand with an Apple pencil and iPad tablet (10.5-inch iPad pro; monitor size, 266.7 mm × 174.11 mm; Apple Inc., Cupertino, CA, USA), as fast and accurately as possible, but not beyond the designated line (Figure 1A). The MT was measured from the time that the pencil made contact with the tablet to when the pencil was returned to the starting position (Figure 1B). The duration was recorded as the MT, which was measured using the tablet. A trial beyond the line (Figure 1C) was considered an error (i.e., no count trial), thus the participant was instructed to perform the task again.

To set different task difficulties, this study set three lines width referring Fitts’ experiments (1954) and as described in previous studies (Vaughan et al., 2010; Hsu and Scholz, 2012). However, because the task was relatively simple, it was unsuitable for motor learning. Therefore, the original circle-drawing task was modified to increase the difficulty of learning the task. The line width of the circle was set at 20 mm (large), 15 mm (medium), and 10 mm (small). The distance between the center of the circle and the midpoint of the line (i.e., radius of the circle) was consistent (*r* = 110 mm; Figure 1A). Therefore, the circumference of the circle (2πr) was approximately 346 mm. The solid black line in Figure 1A was not displayed during all measurements.

#### 2.1.4 Data acquisition and analysis

Before the experiment, the participants performed a few trials by drawing circles on the tablet with the right hand to gain familiarity with all size conditions and avoid errors by drawing beyond the line (Figure 1C). For the actual test, the MTs of 10 successful trials were recorded. The trials were separated by intervals of 10 s (Gentili and Papaxanthis, 2015). The recorded MTs were exported as a csv file and then were analyzed off-line. Data were collected, organized, and analyzed using Unity (application type, Unity technologies, USA), and activated on an iPad using Deploygate (application type, DeployGate Inc. Japan). To calculate the index of difficulty (ID) of the task, based on Fitts’ formula (1954), the line width (i.e., size) was substituted in the equation:


(1) $$\mathrm{ID}=\log2\;\left(2D/W\right)$$

Where *D* is the circumference of the circle (346 mm) and *W* is the width of line (20, 15, or 10 mm).

Moreover, MT was calculated as:


(2) $$MT=a+\left(b\times \mathrm{ID}\right)$$

Where *a* is the constant, *b* is the coefficient, and ID is the index of difficulty. The percentage of errors and the total number of trials were calculated.

#### 2.1.5 Statistical analysis

The data of two participants were excluded because of extremely low MT values (>average plus 3 standard deviations). Therefore, the data of 38 participants were included for analysis. The MT values were compared with one-way repeated measurement analysis of variance (ANOVA) with size (large, medium, and small) as the within-subject factor. The corrected Bonferroni test was to compare the size values. Regression analysis was performed to calculate an equation to predict the MT value, as described above. A probability (*p*) value <0.05 was considered statistically significant. All statistical analyses were performed with IBM SPSS Statistics for Windows (version 20.0; IBM Corporation, Armonk, NY, USA).

### 2.2 Experiment 2

#### 2.2.1 Participants

The study cohort for experiment 2 consisted of 38 healthy young adults, which included some (14) of the participants from experiment 1. Experiment 2 was conducted >1 month after experiment 1. All participants were right-handed without developmental, neurological, mental, or physical. The participants were randomly assigned to the physical training group (9 men and 10 women; mean age, 21.4 ± 1.7 years) or the mental training group (10 males and 9 females; mean age, 21.8 ± 2.3 years).

#### 2.2.2 Experimental procedure and data acquisition

The participants were instructed to draw circles as described for experiment 1. Before testing, the participants were subjected to two or three familiarization trials so that they could draw circles on a tablet with the left (nondominant) hand without drawing beyond the boundary (Figure 1C). The experimental protocol included four sessions: pretest, training, posttest, and retention test. The sessions were conducted at 10-min intervals (Gentili et al., 2006). The trials were conducted at 10-s intervals (Gentili and Papaxanthis, 2015).

##### 2.2.2.1 Pretest session

The participants (*n* = 38) performed the circle-drawing task using the left (nondominant) hand and were instructed to draw as accurately (i.e., not beyond the line) and fast as possible. If the drawing crossed the line, the MT was not recorded and the task was performed again. The MTs of three successful trials (Gentili and Papaxanthis, 2015) were recorded.

##### 2.2.2.2 Training session

In this session, each training modality (physical and mental) was practiced. The participants in the physical training group (*n* = 19) were instructed to draw the medium-sized circle (diameter = 15 mm, blue circle in Figure 1A) using the left (nondominant) hand to determine whether the modality influenced generalization of tasks with different levels of difficulty (i.e., small circle, more difficult; large circle, less difficult). Participants were instructed to draw a circle as accurately and fast as possible in each trial. The participants in this modality group conducted 50 practice trials. The number of practice trials, including error trials (i.e., not counted), was limited to 100, thereby allowing a success rate of only 50%. If the participants performed more than the limited number of trials, a rest time of 3 min was allowed. In total, 6 of 19 participants performed >100 trials during this session. Participants in the mental training group (*n* = 19) simulated drawing with their eyes closed after observing the size of the medium-sized circle. The observation duration was set to >10 s for the participants to imagine the medium-sized circle with their eyes closed. They were instructed to mentally move their arm as accurately and fast as possible, as if physically performing the task. All participants in this group mentally practiced 50 trials. The total practice duration for each modality group was recorded (physical group, 7.9 ± 4.2 min; mental group, 2.8 ± 1.3 min).

##### 2.2.2.3 Posttest session

This session was identical to the pretest session. All participants drew the circle using the left (nondominant) hand and the MTs of three successful trials (Gentili and Papaxanthis, 2015) were recorded.

##### 2.2.2.4 Retention test

This session was identical to the pretest session and conducted 1 day (24 h) after the pretest session (Hamel et al., 2021; Ruffino et al., 2021). All participants drew the circle using the left (nondominant) hand and the MTs of three successful trials (Gentili and Papaxanthis, 2015) were recorded.

#### 2.2.3 Data analysis and statistical analysis

To assess motor adaptation (practiced size, i.e., medium circle) or generalization (nonpracticed sizes, i.e., large and small circles) of left-hand performance in the posttest, the temporal gain between the pretest and posttest was calculated as:


(3) $$\mathrm{gain}=\left(pre-\mathrm{post}\right)/pre\times 100$$

Also, to assess degree of retention after the retention test as compared to the posttest, the retention ratio between the posttest and retention test was calculated as (Ruffino et al., 2021):


(4) $$\mathrm{retention ratio}=\left(\mathrm{post}-\mathrm{retention}\right)/\mathrm{post}\times 100$$

The percentage of errors and the total number of trials in each session were calculated.

Two-way repeated ANOVA with “modality” (mental or physical) as the between-subject factor and “size” (large, medium, or small) as the within-subject factors was performed with the parameters MT of the pretest, gain, and retention ratio. Three-way repeated ANOVA was performed with “modality” as the between-subject factor and “size” and “test” (pre- and posttest or post and retention test) as the within-subject factors: i.e., Test_prepost_ (pretest vs. posttest) and Test_postret_ (posttest vs. retention test). For each ANOVA, the corrected Bonferroni test was performed to examine differences between sizes (large, medium, and small). The level of significance was set at *α* = 0.05. All statistical analyses were performed using IBM SPSS Statistics for Windows (version 20.0).

## 3 Results

### 3.1 Experiment 1

**Figure 2:**
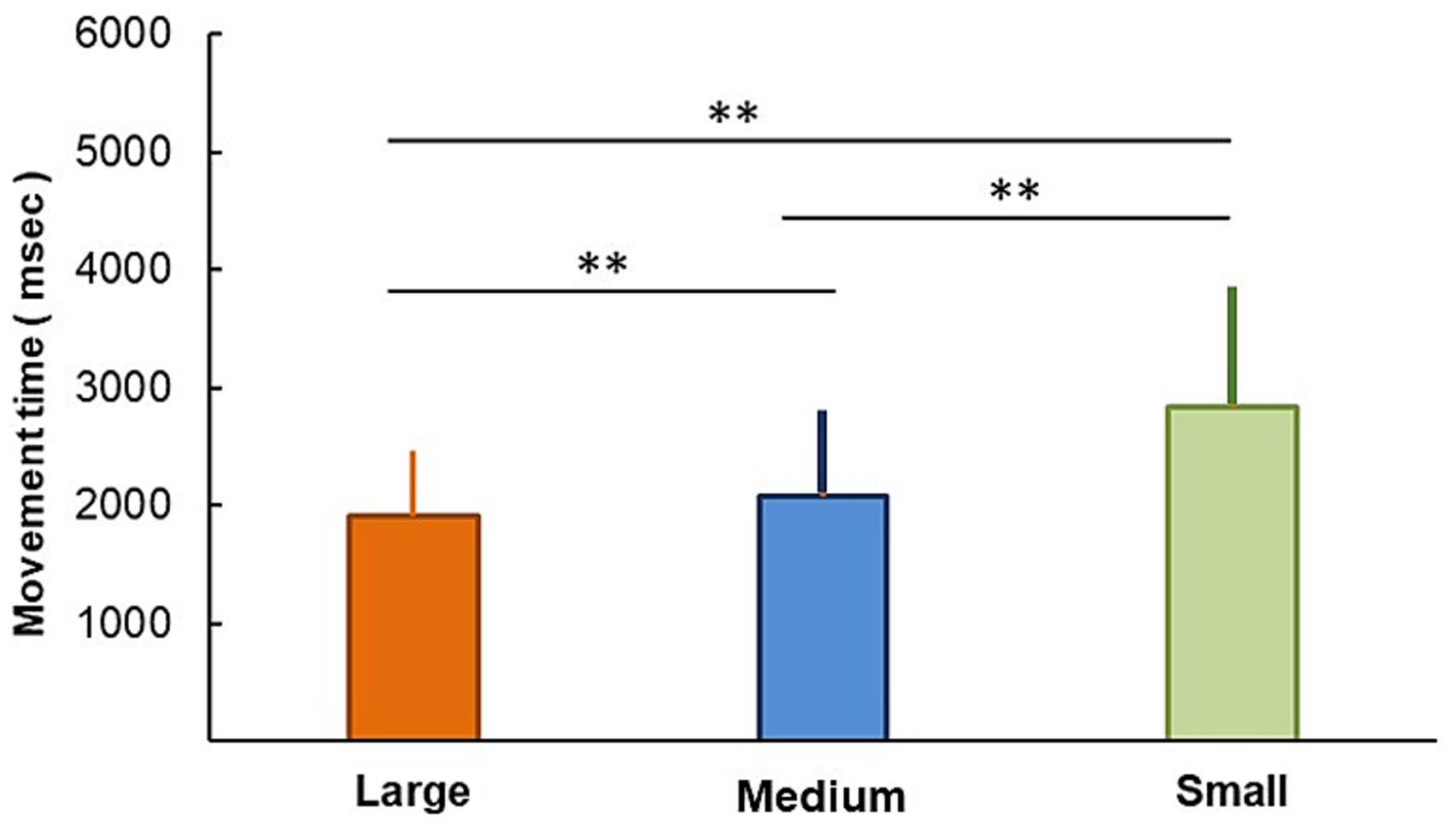
MTs in experiment 1. Averaged (standard deviation) MT for each size. **p < 0.01.

The IDs (Equation 1) for the large, medium, and small circles were 5.113, 5.528, and 6.113, respectively (Table 1). The average MTs ± standard deviations for drawing the large, medium, and small circles were 1,908 ± 558, 2,083 ± 627, and 2,839 ± 949 ms, respectively (Figure 2). The ANOVA results revealed that “size” had a significant effect (*F* (1.37) = 111.44, *p* < 0.001, *η_p_*^2^ = 0.751). The results of the corrected Bonferroni test revealed a significant difference in the MTs among the sizes of the circle, as the MT was shorter for drawing the large circle than the medium (*p* = 0.001) and small (*p* < 0.001) circles. Also, the MT was shorter for drawing the medium circle than the small circle (*p* < 0.001). Table 2 shows the percentage of errors and total trial counts. The minimum percentage of errors was zero under all size conditions, whereas the maximum was 40.0, 100.0, and 190.0 for large, medium, and small circles, respectively. Although some participants had errors exceeding the number of test trials, only zero, one, and five participants for large, medium, and small circles, respectively, exhibited an error percentage of >100 (i.e., >10 errors in 20 trials). Regression analysis revealed that the coefficient *b* and constant *a* (Equation 2) were 954.4 (*p* < 0.001, 95% confidence interval [CI] = 622.045–1,286.811) and − 3,052.8 (*p* = 0.002, 95% CI = −4,913.99 to −1,191.68), respectively, while MT = −3,052 + 954.4 × ID. The ANOVA results showed that this equation accurately predicted the MTs (*F* (1,112) = 32.37, *p* < 0.001). However, as the coefficient of determination (*R*^2^) was 0.224, this equation did not ideally fit the data.

**Table 1:** Index of task difficulty for each “size” condition.

	Large	Medium	Small
ID	5.113	5.528	6.113

ID, Index of task difficulty. Unit is “bit”.

**Table 2:** Percentage of errors and the total number of trials in each size.

		Large	Medium	Small
Percentage of errors	Min (%)	0.0	0.0	0.0
	Max (%)	40.0	100.0	190.0
	>100%	0	1	5
Total number	Min (time)	10	10	10
	Max (time)	14	20	29

>100%: number of participants who exceeded 100 percentages of errors.

Total number: The number of trials including successful three trials.

### 3.2 Experiment 2

The two-way ANOVA results revealed a significant effect of “size” as well as the result of experiment 1 (*F* (1.36) = 146.809, *p* < 0.001, *η_p_*^2^ = 0.803), no significant difference in the average MTs of the mental and physical modality groups in the pretest (*F* (1.36) = 0.054, *p* = 0.818, *η_p_*^2^ = 0.001), and no significant interaction between “size” and “modality” (*F* (1.36) = 0.192, *p* = 0.664, *η_p_*^2^ = 0.005). The results of the corrected Bonferroni test revealed a significant difference in MT among the sizes of the circles. Thus, a change in MT can be interpreted as an effect of each practice trial. The percentage of errors and the total number of trials in each session are shown in Table 3.

**Table 3:** Percentage of errors and the total number of trials in each session.

		Pre						Training	Post						Retention					
		Large		Medium		Small		Medium	Large		Medium		Small		Large		Medium		Small	
		Mental	Physical	Mental	Physical	Mental	Physical	Physical	Mental	Physical	Mental	Physical	Mental	Physical	Mental	Physical	Mental	Physical	Mental	Physical
Percentage of errors	Min (%)	0.0	0.0	0.0	0.0	0.0	0.0	10.0	0.0	0.0	0.0	0.0	0.0	0.0	0.0	0.0	0.0	0.0	0.0	0.0
	Max (%)	100.0	66.7	333.3	266.7	633.7	533.3	196.0	66.7	200.0	133.3	100.0	300.0	400	200.0	66.7	333.3	100	166.7	266.7
	>100%	1	0	3	3	4	2	6	0	1	2	1	4	1	1	0	2	1	6	5
Total number	Min (time)	3	3	3	3	3	3	55	3	0	3	0	3	0	3	3	3	3	3	3
	Max (time)	6	5	10	11	22	19	148	5	9	9	6	12	15	9	5	13	6	8	11

>100%: number of participants who exceeded 100 percentages of errors.

Total number: number of trials including successful three trials.

### 3.3 Comparison of MTs between pretest and posttest after mental and physical training

**Figure 3:**
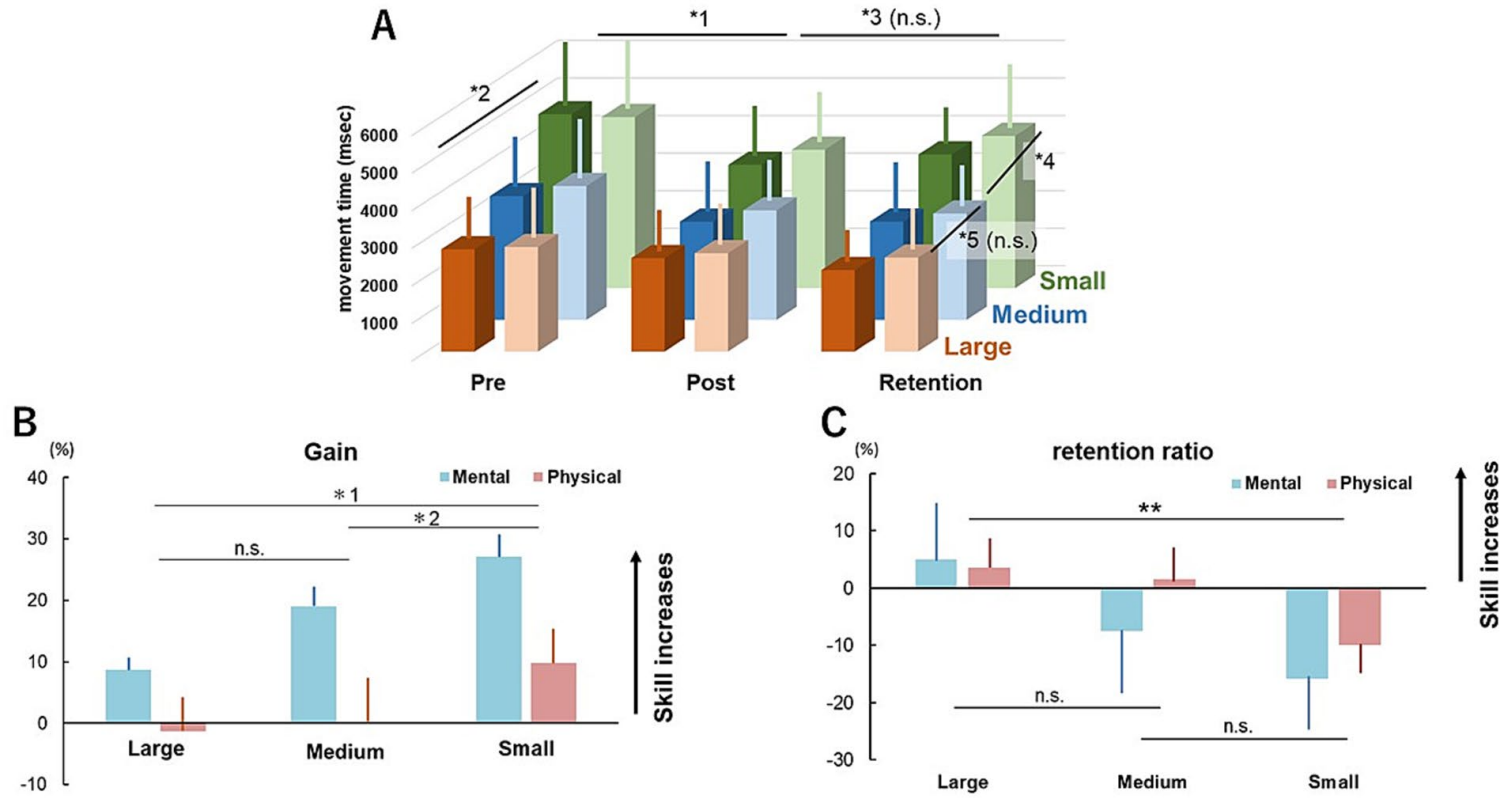
MT, gain, and retention ratio of both modality groups in experiment 2. **(A)** MTs of the mental and physical modality groups of the pretest, posttest, and retention test for the large, medium, and small circles, respectively. Bars indicate standard deviation. The mental and physical modality groups show deep and light colors, respectively, in each size. *1 indicates a significant difference between the pretest and posttest (*p* = 0.006). *2 indicates a significant difference among all sized circles (all *p* < 0.001). *3 indicates no significant difference between the posttest and retention test. *4 indicates a significant difference between the small and medium circles (*p* < 0.001). *5 indicates no significant difference between the medium and large circles. **(B)** Gains of the mental and physical modality groups for each size condition. A positive value indicated improved performance. Bars indicate standard deviation. *1 indicates *p* < 0.001 for small vs. large. *2 indicates *p* = 0.004 for small vs. medium. n.s. = not significant. **(C)** Retention ratios of the mental and physical groups for ‘each size condition’. A positive value indicates improved performance. Bars indicate the standard error. **p = 0.001 for small vs. large. n.s. = not significant.

MTs of mental and physical training were shorter for the posttest than the pretest (Figure 3A). The three-way ANOVA results revealed a significant interactive effect between “Test_prepost_” and “size” (*F* (1.72) = 27.223, *p* < 0.001, *η_p_*^2^ = 0.274), which was a larger reduction of MT for the small circle than that for large circle, and a significant effect of “Test _prepost_” (*F* (1.72) = 7.929, *p* = 0.006, *η_p_*^2^ = 0.099). The results of the corrected Bonferroni test revealed a significant difference in MT among all sizes (all *p* < 0.001), as MT was significantly longer with decreasing size (Figure 3A). In contrast, there was no significant interactive effect between “size” and “modality” (*F* (1.72) = 0.152, *p* = 0.698, *η_p_*^2^ = 0.002), between “modality” and “test” (*F* (1.72) = 0.17, *p* = 0.681, *η_p_*^2^ = 0.002), among “size,” “modality” and “test” (*F* (1.72) = 1.334, *p* = 0.252, *η_p_*^2^ = 0.018), and no significant effect of “modality” (*F* (1.72) = 0.603, *p* = 0.440, *η_p_*^2^ = 0.008). The percentage of errors and total number of trials in each group in the pretest and posttest are shown in Table 3. Although for some participants the errors exceeded the number of test trials, only 0–4 of the 19 participants in each group exhibited an error percentage of >100 (i.e., >3 trials in 6 trials) in the pre- and posttest sessions.

The gains (Equation 3) in each size for both modality groups are shown in Figure 3B. The results of two-way ANOVA for the gain revealed no significant interactive effect between “size” and “modality” (*F* (1.36) = 1.561, *p* = 0.220, *η_p_*^2^ = 0.042), but significant individual effects of “modality” (*F* (1.36) = 6.407, *p* = 0.016, *η_p_*^2^ = 0.151) and “size” (*F* (1.36) = 25.147, *p* < 0.001, *η_p_*^2^ = 0.411). The gain was significantly larger for the mental training group than the physical training group. The results of the corrected Bonferroni test revealed that the gain was larger for “small” than “large” and “medium” (small vs. large: *p* < 0.001, 95% CI = 7.374–22.166; small vs. medium: *p* = 0.004, 95% CI = 2.381–15.237), but no significant difference between “large” and “medium” (*p* = 0.102, 95% CI = −12.753 to 0.831).

### 3.4 Comparisons of MTs between the posttest and retention test after mental and physical training

The MTs were similar for the retention test and posttest (Figure 3A). The results of three-way ANOVA revealed no significant interactive effect between “modality” and “Test_postret_” (*F* (1.72) = 0.028, *p* = 0.867, *η_p_*^2^ = 0.001), between “modality” and “size” (*F* (1.72) = 2.737, *p* = 0.102, *η_p_*^2^ = 0.037), among “size,” “modality” and “test” (*F* (1.72) = 0.174, *p* = 0.678, *η_p_*^2^ = 0.002) or individually for “modality” (*F* (1.72) = 2.262, *p* = 0.137, *η*_p_^2^ = 0.030) and “Test_postret_” (*F* (1.72) = 0.01, *p* = 0.921, *η_p_*^2^ = 0.000). In contrast, there was significant interactive effect between “Test_postret_” and “size” (*F* (1.72) = 17.277, *p* < 0.001, *η_p_*^2^ = 0.194), which was lower retention ratio for the small circle than the large circle, and individually for “size” (*F* (1.72) = 343.649, *p* < 0.001, *η_p_*^2^ = 0.827). The results of the corrected Bonferroni test revealed that the MT was longer for “small” than “medium” (*p* < 0.001, 95% CI = 540.996–1,263.654) and “large” (*p* < 0.001, 95% CI = 841.029–1,563.687), while there was no significant difference between “medium” and “large” (*p* = 0.125, 95% CI = −61.296 to 661.362). The percentage of errors and total number of trials in each group in the retention test are shown in Table 3. Although for some participants the errors exceeded the number of test trials, only 0–6 of the19 participants exhibited an error percentage of >100 (i.e., >3 trials in 6 trials) in each group in the pre- and posttest sessions.

The retention ratios (Equation 4) are shown in Figure 3C. The results of two-way ANOVA revealed a significant individual effect of effect of “size” (*F* (1.36) = 17.021, *p* < 0.001, *η_p_*^2^ = 0.321), but not “modality” (*F* (1.36) = 0.217, *p* = 0.644, *η_p_*^2^ = 0.006). The results of the corrected Bonferroni test revealed that the retention ratio was significantly smaller for “small” than “large” (*p* = 0.001, 95% CI = −27.65 to −6.727), while there was no significant difference in the retention ratio of “medium” as compared to “small” and “large” (medium vs. small: *p* = 0.077, 95% CI = −0.764 to 20.487; medium vs. large: *p* = 0.085, 95% CI = −15.382 to 0.727).

## 4 Discussion

### 4.1 Experiment 1

The circumferences were consistent among circles of the same size (Figure 1A) to allow the participants to draw the circles with the same duration. However, since the participants were instructed to draw circles as accurately and fast as possible, the MTs became longer with narrowing line widths with notable difference among the three different sizes (Figure 2). These differences may have resulted from greater accuracy of arm movements while drawing circles with narrower widths. As expected from Fitts’ law (1954, 1965), accuracy was improved with narrowing widths of the circles, while the MTs increased. Therefore, the differences in circle widths indicate differences in task difficulty as well as Fitts’ task.

Based on Fitts’ equations (1964), the predictive equation for MT were calculated using regression analysis. The predictive equation for the MTs was based on extended tasks as well as Fitts’ original task (Jax et al., 2007; Vaughan et al., 2010). Tasks in previous studies, including Fitts’ original task, were three-dimensional motions to touch two plates with a tool. The present task was a two-dimensional motion, which involved drawing a circle on a tablet. Therefore, since the differences in the dimensions of these tasks might have influenced the results (Murata and Iwase, 2001), it was necessary to determine whether a predictive equation can predict MT. The ANOVA results showed a significant coefficient and constant, indicating that the MTs could be predicted based on the IDs calculated by the circle widths (i.e., size). In contrast, because the determination coefficient was not high, the equation was carefully fit to actual data. The lack of a high determination coefficient may be attributed to the large variance in MTs among the participants for each size. A previous study reported that more complex tasks are associated with larger variances among participants, resulting in a lower determination coefficient of the predictive equation for MT (*R*^2^ = 0.31, Vaughan et al., 2010). This determination coefficient was similar to that in the present study. Nonetheless, drawing a circle is not a complex motion, but might be more complex than a reaching motion or movement between two plates, such as Fitts’ original task. Furthermore, if there were errors with the present task (i.e., drawing beyond the line), the trial was performed again. Therefore, some participants might be more careful to avoid errors, although they were instructed to draw as fast as possible, which possibly could explain the large variation in the MTs and relatively low determination coefficient. The large variation in the MTs might have been influenced by the arm movements of the participants. Hence, future research should focus not only on MT, but also body kinematics, to assess motor adaptation in terms of intrinsic variables, such as body kinematics.

### 4.2 Experiment 2

This study investigated the three following topics in terms of the effects of mental and physical training on motor learning using the original reciprocal tapping task described by Fitts: (i) whether the immediate effects of the two modalities result in performance differences, (ii) whether the difficulty of the task is associated with differences in immediate generalization of the two modalities, and (iii) whether the degrees of retention and generalization differ between the two modalities.

#### 4.2.1 Differences in immediate effects between mental and physical training

The MTs of the posttest with both modalities were shorter than the pretest. This difference indicates that both modalities could positively impact immediate motor adaptation or generalization. This finding is supported by previous studies (Gentili and Papaxanthis, 2015; Grèzes and Decety, 2001; Ruffino et al., 2017; Sirigu et al., 1996). Also, the present study showed that gain was larger for the mental training group than the physical training group, indicating that mental simulation can have a greater immediate effect on performance than actual training. The total number of trials in the pretest was slightly higher for the mental training group than the physical training group (Table 3), potentially influencing the results and producing higher gains for the mental training group. Future studies should ensure an equal number of trials between mental and physical training groups, conducted without task error. Since the participants performed the task using the nondominant hand, which can be controlled by feedback-mediated mechanisms (Mutha et al., 2013), mental simulation with no sensory feedback might diminish the effects of training than actual training with sensory feedback. However, the present finding contradicted the hypothesis, and differed from the results reported previously, in which MT was recorded using its original task in physical and mental training groups, revealing a more substantial reduction in MT in the former than in the latter (Gentili and Papaxanthis, 2015). The difference between the present and previous results could be attributed to the type of task in the current study. Performance of the present task was considered incorrect if the circle was drawn beyond the line, thus the MT was not recorded and the participant was instructed to repeat the task (Figure 1C). During the practice session, most participants in the physical training group had to repeat the task at least once. This error is not an implicit recalibration modulated by sensory prediction errors but rather the movement goal, known as a task error. Previous studies have reported that sensory prediction errors can drive motor adaptation (Izawa and Shadmehr, 2011; Marko et al., 2012; Palidis et al., 2021; Popa et al., 2016). In contrast, another study reported that task errors without sensory prediction errors were unable to drive motor adaptation, thus failing implicit recalibration (Tsay et al., 2022). Specifically, this would be the kind of error that can affect success or failure in motor adaptation. The finding that the gain was larger with mental training than with actual practice may be attributed to task errors. There were task errors in the physical training group. Conversely, there was no task error in the mental training group owing to no actual movements. Therefore, since mental simulation without task errors promotes motor adaptation, the gain was larger for the mental training group than the physical training group. The MT of the posttest for both modality groups was faster than the pretest, as simulations with an internal forward model are more common for mental and physical training (Gentili and Papaxanthis, 2015). In addition, a task error can result in differences in mental and physical training effects. Therefore, future studies should focus on clarifying the effects of task errors.

#### 4.2.2 Generalization of mental and physical training with different task difficulties

This study examined how training with the medium-sized circle can be immediately generalized to the easier task with the larger circle and the more difficult task with the smaller circle. The results of this study found no significant interaction between “modality” and “size,” while “Test_prepost_” had a significant influence, indicating that there was no difference in the degree of immediate generalization between the modality groups. Notably, gain was significantly larger for the small circle than the large and medium circles, while there was no significant difference in gain between the large and medium circles. These findings indicate that immediate generalization can occur with both modalities, thus training with the medium-sized circle can improve MT with the small circle without practice. Furthermore, generalization might be greater with more difficult tasks.

Previous studies have reported that actual training can improve motor learning (Sainburg et al., 1999; Scheidt et al., 2000; Thoroughman and Shadmehr, 2000). However, no study has investigated the effects of mental training on motor learning (generalization) in terms of different task difficulties. The findings of the present study are novel, and may highlight reinforcement and update of internal forward models. The finding that generalization is dependent on the difficulty of the task might be explained by the framework proposed by Guadagnoli and Lee (2004), which suggests that optimal learning is associated with the difficulty of a task. Because the large-sized condition was easy for participants, it may not be an optimal learning task for generalization, resulting in a low degree of generalization under this condition. In contrast, the small-sized condition, which had an appropriate level of difficulty for participants, it may be an optimal learning task for generalization. Overall, the degree of generalization under the small-sized condition would be higher than that under the large-sized one.

#### 4.2.3 Retention with mental and physical training for different task difficulties

There were no significant differences in MTs with either modality between the retention test and the posttest (Figure 3A), indicating that immediate motor adaptation and generalization with both modalities in the posttest were maintained after 24 h, in agreement with the findings of previous studies, which reported persistent changes to motor behaviors (Debarnot et al., 2009; Ruffino et al., 2021). A paradigm of visuomotor adaptation suggests that the retention interval can positively affect consolidation of the learning process (Krakauer et al., 2005). In addition, both physical training and mental training have been linked to consolidation during sleep (Debarnot et al., 2009). Moreover, learning a spatial movement (i.e., MT in the present study) can be reinforced while sleeping rather than when awake (Cohen et al., 2005). In the present study, the retention test was conducted 24 h after the posttest. Therefore, there was a period of sleep between the two tests. The goal of the task in the present study was to shorten the MT as fast as possible. After the posttest, performance was not associated with reinforcement of retention in the retention test, suggesting that the goal of improved MT by mental and physical training was not achieved by sleeping. Although an explicit factor is reportedly reinforced during sleep, it remains unclear whether consolidation results from explicit factors (e.g., MT) or implicit factors (e.g., joint coordination). Therefore, further studies are needed to assess the effects of implicit factors, such as joint coordination, on tasks, such as drawing a circle, as described in the present study.

Interestingly, there was a significant interaction between “size” and “Test_postret_,” and a significant effect of “size” on the retention ratio. Notably, the retention ratio was significantly lower for the small circle than the large circle (Figure 3C). As shown in Figure 3A, the retention ratios of the small and large sizes had positive and negative values, respectively, with both modalities, indicating longer and shorter MTs than with the posttest. Thus, immediate generalization of a more difficult task might be more unlikely than with an easier task. In this study, training was performed with the medium-sized circle to assess the degree of retention with tasks with different levels of difficulty. Moreover, training was performed with the small circle to assess retention with a more difficult task and with the large circle to assess generalization to a less difficult task. The results showed that generalization of more difficult tasks was less likely to be retained, regardless of the modality, which was inconsistent with the hypothesis and the findings of previous studies that physical training can more effectively induce neurological changes related to motor learning than mental training (Allami et al., 2008; Allami et al., 2014; Kraeutner et al., 2020). This inconsistency may be attributed to the task in the present study. As task errors inhibit implicit recalibration (Tsay et al., 2022), physical training, which occurs task errors during training session unlike mental training, did not result in appropriate calibration. Therefore, physical training does not necessarily improve performance as compared to mental training, as demonstrated in the present study. Although there was no clear difference between the modalities, generalization for tasks with low difficulty (i.e., large size in the present study) was more effectively retained and reinforced than tasks with high difficulty. This finding is innovative as no prior study had compared mental and physical training in terms of retention of tasks with high vs. low difficulty.

There were some limitations in this study. First, this study did not assess the participants’ ability to perform a kinesthetic imagery task such as a movement imagery questionnaire (MIQ). Because the mental training group performed a kinesthetic imagery task, the ability might affect the effect of mental training. However, a previous study (Ruffino et al., 2021) enrolled younger individuals with a wide range of various ability (minimum and maximum MIQ scores of 8 and 56, respectively) as participants. The previous study described that enrollment did not influence the effect of mental training. Considering the ability could not affect the present results, this study did not assess the accuracy of mental imagery. Second, due to task errors, the total number of training trials differed between the two modality groups, potentially causing a lack of isochrony between the mental training and required task. Although the difference in the total number of trials might influence the effect of both trainings, the difference of the total number of trials in the pretest between the two modality groups was only two. Therefore, the difference would not affect the results. Third, the larger gain for the mental training group compared to the physical training group may be influenced by the slightly higher total number of trials in the pretest for the mental training group. Although a few more trials between the mental and physical training groups would not affect the gain difference, future studies should ensure the same number of trials between groups without task error. Fourth, spacing parameters such as the trajectory length of the task were not measured. A longer trajectory length might lead to longer MTs, potentially influencing the results. However, previous studies based on Fitts’ law (e.g., Fitts, 1954) reported that the difficulty level changed with changing line width, regardless of the modifications in the distance of the hand movement. Thus, to interpret the results of the present study based on previous studies, it was inferred that the difference in MTs was influenced by the line width, indicating a difference in difficulty level.

In conclusion, this study examined the effects of mental and physical training on immediate generalization and retention of a drawing task with different levels of difficulty in reference to Fitts’ law (Fitts, 1954; Fitts and Peterson, 1964). The results found that immediate generalization was associated with larger gains by mental training rather than physical training regardless of the difficulty of the task, while the training modality had no significant effect on retention, although retention was influenced by the difficulty of the task. Future studies are warranted to assess not only explicit parameters (i.e., MT), but also implicit parameters (e.g., joint movement patterns), to clarify the degrees of generalization and retention of complex tasks.

## Data Availability

The original contributions presented in the study are included in the article/Supplementary material, further inquiries can be directed to the corresponding author.
