# Supplementary material for: Effects of motor imagery training on generalization and retention for different task difficulties
Source: Front Hum Neurosci. 2024 Oct 16;18:1459987. doi: 10.3389/fnhum.2024.1459987 (PMC11521821; doi:10.3389/fnhum.2024.1459987)
Supplement: Supplementary file 1 [file Table_1.DOCX]

Supplementary Material

**sTable 1. Raw data in experiment 1**

| Participant No. | Large | Middle | Small |  |
| --- | --- | --- | --- | --- |
| 1 | 1478.7 | 1610.7 | 1955.3 |  |
| 2 | 2244.7 | 2377.0 | 3444.0 |  |
| 3 | 3077.0 | 2843.7 | 3399.3 |  |
| 4 | 2533.7 | 2955.0 | 4432.7 |  |
| 5 | 1399.3 | 1922.0 | 3405.0 |  |
| 6 | 1621.7 | 1755.0 | 2676.7 |  |
| 7 | 2277.3 | 2321.0 | 3232.3 |  |
| 8 | 2499.3 | 2521.7 | 3988.3 |  |
| 9 | 2455.0 | 2846.0 | 3923.0 |  |
| 10 | 1533.7 | 1899.3 | 2300.0 |  |
| 11 | 2466.3 | 2932.7 | 3766.0 |  |
| 12 | 1732.3 | 1843.7 | 2399.3 |  |
| 13 | 1988.3 | 2410.3 | 3864.0 |  |
| 14 | 2221.3 | 2800.0 | 3400.0 |  |
| 15 | 1532.7 | 1899.3 | 2121.3 |  |
| 16 | 1678.7 | 1643.3 | 1910.3 |  |
| 17 | 2332.0 | 2655.0 | 2899.3 |  |
| 18 | 3078.7 | 3265.7 | 4342.7 |  |
| 19 | 2132.7 | 1987.7 | 2710.3 |  |
| 20 | 1877.7 | 1989.0 | 3288.0 |  |
| 21 | 2465.7 | 2388.0 | 3431.3 |  |
| 22 | 2188.7 | 2100.7 | 3155.0 |  |
| 23 | 2333.7 | 2944.0 | 4088.3 |  |
| 24 | 1477.0 | 1400.7 | 1711.7 |  |
| 25 | 1910.3 | 1688.3 | 2844.7 |  |
| 26 | 3222.0 | 3799.3 | 5421.3 |  |
| 27 | 1288.3 | 1511.3 | 1835.0 |  |
| 28 | 1266.0 | 1566.0 | 1688.7 |  |
| 29 | 1223.8 | 1905.2 | 2449.9 |  |
| 30 | 1328.7 | 1437.9 | 2010.6 |  |
| 31 | 1156.9 | 1260.1 | 1401.1 |  |
| 32 | 1569.3 | 1604.0 | 2334.3 |  |
| 33 | 1585.6 | 1637.0 | 1925.8 |  |
| 34 | 1315.2 | 1710.9 | 2270.9 |  |
| 35 | 1323.4 | 1326.6 | 1727.0 |  |
| 36 | 1694.0 | 1300.2 | 1985.0 |  |
| 37 | 1635.9 | 1730.6 | 2046.6 |  |
| 38 | 1387.9 | 1364.1 | 2112.3 |  |
| 39 | 4222.7 | 4977.0 | 6088.0 | Excluded |
| 40 | 4666.0 | 4854.7 | 7154.7 |  |

**sTable 2. Raw data of pretest in experiment 2**

| Participant No. | Pretest | | | | | |
| --- | --- | --- | --- | --- | --- | --- |
|  | Mental Group | | | Physical Group | | |
|  | Large | Middle | Small | Large | Middle | Small |
| 1 | 1900.0 | 2677.3 | 2989.3 | 3277.7 | 5099.3 | 5800.3 |
| 2 | 2700.7 | 3121.7 | 4288.0 | 2843.7 | 4933.7 | 4810.3 |
| 3 | 3410.7 | 4955.3 | 4532.7 | 2999.3 | 5018.7 | 5167.0 |
| 4 | 3610.3 | 4055.3 | 6599.0 | 3133.7 | 3766.0 | 5255.0 |
| 5 | 1588.3 | 2644.0 | 4132.3 | 2367.0 | 2844.0 | 3054.7 |
| 6 | 2577.0 | 3567.3 | 4710.3 | 1966.0 | 2788.3 | 4354.7 |
| 7 | 3522.7 | 3088.3 | 4977.0 | 6166.7 | 6466.0 | 8021.7 |
| 8 | 2232.3 | 3155.0 | 6977.3 | 1522.0 | 1988.3 | 2610.7 |
| 9 | 4210.7 | 5788.0 | 5910.7 | 1343.7 | 2077.0 | 1723.0 |
| 10 | 3032.7 | 4299.0 | 7833.0 | 1943.7 | 2100.0 | 3366.0 |
| 11 | 1677.3 | 2011.3 | 2244.0 | 2409.3 | 2778.7 | 3906.7 |
| 12 | 2210.3 | 1899.0 | 3077.3 | 2499.3 | 3022.7 | 4077.3 |
| 13 | 2343.7 | 3077.0 | 4477.0 | 3377.0 | 4621.3 | 5632.7 |
| 14 | 4221.3 | 4366.0 | 5765.7 | 2544.0 | 3259.0 | 4699.3 |
| 15 | 2510.3 | 2876.7 | 3643.7 | 2543.7 | 3000.0 | 4201.3 |
| 16 | 2632.7 | 2821.7 | 4343.3 | 1844.3 | 2110.3 | 2789.3 |
| 17 | 2969.7 | 3543.7 | 4254.7 | 2510.3 | 2954.7 | 5176.7 |
| 18 | 2032.3 | 2065.7 | 3488.3 | 2777.7 | 3577.0 | 4899.0 |
| 19 | 2377.0 | 2598.3 | 3744.3 | 4832.3 | 5299.0 | 7055.0 |

**sTable 3. Raw data of posttest in experiment 2**

| Participant No. | Posttest | | | | | |
| --- | --- | --- | --- | --- | --- | --- |
|  | Mental Group | | | Physical Group | | |
|  | Large | Middle | Small | Large | Middle | Small |
| 1 | 1616.5 | 1711.7 | 2099.3 | 3332.5 | 2877.3 | 3555.0 |
| 2 | 2682.5 | 2532.7 | 3067.0 | 2649.5 | 3433.7 | 5032.3 |
| 3 | 2499.5 | 2866.3 | 3199.3 | 2349.5 | 3177.0 | 3377.0 |
| 4 | 3815.5 | 3377.7 | 5138.3 | 2633.0 | 3155.0 | 3632.7 |
| 5 | 1549.5 | 1877.0 | 2521.3 | 1899.0 | 2010.7 | 2500.0 |
| 6 | 2515.5 | 2599.3 | 3577.3 | 2383.5 | 2988.3 | 3877.3 |
| 7 | 2749.0 | 2767.0 | 3144.3 | 4433.0 | 5020.0 | 6221.3 |
| 8 | 2234.5 | 2354.7 | 2632.7 | 1733.0 | 1944.3 | 2879.3 |
| 9 | 4334.0 | 4954.7 | 6632.3 | 2132.5 | 2344.0 | 2989.0 |
| 10 | 2532.5 | 2721.7 | 3332.7 | 1683.0 | 2077.0 | 3332.7 |
| 11 | 1632.5 | 1810.3 | 2021.3 | 2933.5 | 2622.0 | 3511.0 |
| 12 | 1966.0 | 1955.0 | 2399.3 | 2166.0 | 2366.0 | 3021.7 |
| 13 | 2184.5 | 2010.3 | 3132.0 | 2599.0 | 3599.0 | 3888.3 |
| 14 | 3816.0 | 4110.3 | 4410.3 | 2383.0 | 2122.0 | 2299.3 |
| 15 | 2168.0 | 2310.3 | 2488.3 | 2666.5 | 2754.7 | 3599.7 |
| 16 | 2367.5 | 2477.0 | 3077.0 | 1849.5 | 2189.3 | 2644.7 |
| 17 | 2899.5 | 2777.3 | 3911.0 | 3098.5 | 3531.0 | 4776.3 |
| 18 | 1732.5 | 2328.7 | 2732.7 | 2065.5 | 2410.3 | 3088.0 |
| 19 | 1983.0 | 2144.0 | 2932.3 | 4783.0 | 4788.0 | 5822.3 |

**sTable 4. Raw data of retention test in experiment 2**

| Participant No. | Retention test | | | | | |
| --- | --- | --- | --- | --- | --- | --- |
|  | Mental Group | | | Physical Group | | |
|  | Large | Middle | Small | Large | Middle | Small |
| 1 | 1666.3 | 1844.0 | 3144.0 | 1666.3 | 1844.0 | 3144.0 |
| 2 | 2177.0 | 2610.7 | 4033.7 | 2177.0 | 2610.7 | 4033.7 |
| 3 | 2278.7 | 2899.3 | 4443.3 | 2278.7 | 2899.3 | 4443.3 |
| 4 | 2921.3 | 2966.3 | 4844.0 | 2921.3 | 2966.3 | 4844.0 |
| 5 | 1678.0 | 2076.7 | 2777.0 | 1678.0 | 2076.7 | 2777.0 |
| 6 | 2023.7 | 2399.0 | 3043.7 | 2799.7 | 3032.7 | 4088.0 |
| 7 | 2266.0 | 2544.0 | 3566.3 | 1788.0 | 1942.7 | 2543.3 |
| 8 | 1554.7 | 2299.3 | 3344.0 | 2312.0 | 2578.0 | 4288.3 |
| 9 | 2399.3 | 2821.7 | 3421.3 | 2688.7 | 2721.3 | 3921.3 |
| 10 | 1465.7 | 1622.0 | 2577.0 | 4743.7 | 4966.7 | 7688.3 |
| 11 | 2177.0 | 2388.0 | 3688.0 | 2632.7 | 2821.7 | 4343.3 |
| 12 | 2878.0 | 2933.0 | 4077.3 | 2969.7 | 3543.7 | 4254.7 |
| 13 | 2977.3 | 4600.0 | 4944.7 | 2032.3 | 2065.7 | 3488.3 |
| 14 | 1399.7 | 1943.7 | 3943.3 | 2377.0 | 2598.3 | 3744.3 |
| 15 | 1454.7 | 1589.0 | 2076.0 | 2067.0 | 2377.0 | 2610.3 |
| 16 | 1675.7 | 2043.7 | 2521.7 | 2021.3 | 2455.0 | 3590.0 |
| 17 | 2232.7 | 2866.0 | 4143.7 | 4733.3 | 5511.3 | 7499.3 |
| 18 | 3910.7 | 4800.0 | 4221.3 | 2300.0 | 3255.7 | 3621.3 |
| 19 | 2077.0 | 2478.7 | 2845.0 | 1366.0 | 1532.7 | 2122.3 |
